# A Case of Salivary Calculus

**Published:** 1877-07

**Authors:** 


### A CASE OF SALIVARY CALCULUS.

The following interesting case is given in the *British Medical Journal*, by Dr. Reid, of Aberdeen. A man between thirty and forty years of age, otherwise in good health, had been troubled for several months with pain and swelling in the region of the submaxillary gland. But little swelling was preceptible between the gland and the outline of the duct, although there was swelling in the gland itself. Liniments, poultices, warm water kept in the mouth, etc., were resorted to without any relief, and the pain and stiffening of the jaw became increased, so that swallowing was scarcely possible. A swelling beneath the tongue began to appear at this time, and, in the course of two or three days from the first appearance of this swelling, the duct being much distended, a whitish speck was seen in the sublingual caruncula; and on an opening being made, a concretion about the size of a pea was easily scooped out. The operation relieved the pain entirely, but the swelling did not altogether subside; and a relapse took place in about a year. The concretion in this case, though small, was large enough to close up the duct and obstruct the passage of the secretion, and caused a swelling like a half developed ranula. Had the tumor been opened at the thinnest part, the concretion would most likely have escaped observation. The case seemed to indicate that the mere size of the concretion did not determine the extent of the dilatation of the duct, but that it acted, whether large or small, as an obstruction, and might cause enlargement to any extent almost. The calculi in both the cases consisted of phosphate of lime with a little organic matter. Dr. Reid brought out the case on account of the analogy which seemed to him to exist between the formation of these concretions in the glands, their escape, and their slow and painful passage along ducts, and the urinary deposits in the pelvis of the kidney, their escape, and passage along the ureters.—*Medical and Surgical Reporter.*

